# Doxorubicin‐induced senescence promotes stemness and tumorigenicity in EpCAM−/CD133− nonstem cell population in hepatocellular carcinoma cell line, HuH‐7

**DOI:** 10.1002/1878-0261.12916

**Published:** 2021-03-08

**Authors:** Mustafa Karabicici, Sena Alptekin, Zeynep Fırtına Karagonlar, Esra Erdal

**Affiliations:** ^1^ Izmir Biomedicine and Genome Center Turkey; ^2^ Genetics and Bioengineering Department Izmir University of Economics Turkey; ^3^ Department of Medical Biology and Genetics Faculty of Medicine Dokuz Eylul University Izmir Turkey; ^4^ Present address: Department of Internal Medicine III University of Ulm Albert‐Einstein‐Allee 23 Ulm 89081 Germany

**Keywords:** cancer stem cell, EpCAM, hepatocellular carcinoma, therapy‐induced senescence, WNT

## Abstract

The therapeutic induction of senescence is a potential means to treat cancer, primarily acting through the induction of a persistent growth‐arrested state in tumors. However, recent studies have indicated that therapy‐induced senescence (TIS) in tumor cells allows for the prolonged survival of a subgroup of cells in a dormant state, with the potential to re‐enter the cell cycle along with an increased stemness gene expression. Residual cells after TIS with increased cancer stem cell phenotype may have profound implications for tumor aggressiveness and disease recurrence. Herein, we investigated senescence‐associated stemness in EpCAM+/CD133+ liver cancer stem cell and EpCAM−/CD133− nonstem cell populations in HuH7 cell line. We demonstrated that treatment with doxorubicin induces senescence in both cell populations, accompanied by a significant increase in the expression of reprogramming genes SOX2, KLF4, and c‐MYC as well as liver stemness‐related genes EpCAM, CK19, and ANXA3 and the multidrug resistance‐related gene ABCG2. Moreover, doxorubicin treatment significantly increased EpCAM + population in nonstem cells indicating senescence‐associated reprogramming of nonstem cell population. Also, Wnt/β‐catenin target genes were increased in these cells, while inhibition of this signaling pathway decreased stem cell gene expression. Importantly, Dox‐treated EpCAM−/CD133− nonstem cells had increased in vivo tumor‐forming ability. In addition, when SASP‐CM from Dox‐treated cells were applied onto hİPSC‐derived hepatocytes, senescence was induced in hepatocytes along with an increased expression of TGF‐β, KLF4, and AXIN2. Importantly, SASP‐CM was not able to induce senescence in Hep3B‐TR cells, a derivative line rendered resistant to TGF‐β signaling. Furthermore, ELISA experiments revealed that the SASP‐CM of Dox‐treated cells contain inflammatory cytokines IL8 and IP10. In summary, our findings further emphasize the importance of carefully dissecting the beneficial and detrimental aspects of prosenescence therapy in HCC and support the potential use of senolytic drugs in HCC treatment in order to eliminate adverse effects of TIS.

## Graphical abstract

In this study, we showed that therapy‐induced senescence promotes tumorigenicity and stemness in liver cancer cells. Doxorubicin treatment induced senescence in both EpCAM+/CD133+ liver cancer stem cells and EpCAM−/CD133− nonstem cells. Doxorubicin‐treated EpCAM−/CD133− cells had higher expression of stemness‐related genes and displayed higher tumor‐initiating capacity than their *non‐senescent counterparts*. Notably, conditioned media from senescent doxorubicin‐treated EpCAM−/CD133− nonstem cells induced senescence and stemness in healthy hepatocytes.

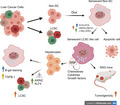

**Abbreviations**
Dox: doxorubicin
HCC: hepatocellular carcinoma
iPSCs: inducible pluripotent stem cells
LCSC: liver cancer stem cell
NSG: NOD/SCID/GAMMA
SASP: senescence‐associated secretory phenotype
SASP‐CM: SASP‐conditioned media
TIS: therapy‐induced senescence

## 1 Introduction

Hepatocellular carcinoma (HCC) remains difficult to treat, owing to its complex and heterogeneous nature. As a result of advanced disease state and underlying poor liver function at the time of prognosis, only few patients with HCC are eligible for surgery and the current systemic treatment agents (sorafenib, lenvatinib, regorafenib, and others) do not offer an increase in the average survival time over 3–4 months [1, 2]. The therapeutic induction of senescence, especially followed by senolytic agents which selectively induce removal of senescent cells, holds hope for a new potential treatment strategy.

Therapy‐induced senescence (TIS) can be induced by cancer therapies such as cytotoxic therapies, molecularly targeted therapies, and immunotherapies [3, 4, 5, 6]. Although both apoptosis and senescence represent cell cycle exit programs that can be induced by therapeutic agents, apoptotic cell death is quantitatively more prominent tumor response. However, in contrast to apoptotic cells, senescent cells remain viable for an extended time and often exhibit senescence‐associated secretory phenotype (SASP) that can have important long‐term implications on the treatment outcome [7, 8, 9]. In addition to cell‐autonomous senescent phenotype of the tumor cell, SASP involves the secretion of a wide range of cytokines, chemokines, and growth factors that affect surrounding cells and compartments leading to noncell‐autonomous phenotypes at the tumor site [9, 10, 11]. Through this phenotype, senescent cells have an immerse effect that might be beneficial or deleterious for the tumor microenvironment [12, 13].

Importantly, recent studies indicate an increase in stem cell features of cancer cells undergoing therapy‐induced senescence. The so called ‘senescence‐associated reprogramming’ of a subgroup of tumor cells into cancer stem cell‐like state has been suggested by different groups [14, 15, 16, 17]. Recently, Milanovic et al. (2018) established a switchable model for therapy‐induced senescence using Eμ‐Myc transgenic Bcl2‐overexpressing lymphomas to unmask the tumorigenicity of cells that acquired senescence‐associated stemness. In this model, they demonstrated that senescence‐reprogrammed cells with increased levels of stem cell‐related transcripts show enhanced tumor‐initiating potential compared with never senescent cells after their forced release or spontaneous escape from a chemotherapy‐induced senescent cell cycle arrest [18]. Their results propose that some features such as stemness acquired during senescence might still be propagated in postsenescent cells. More importantly, their data imply that the senescent state can be reversible when senescence regulation and/or maintenance genes such as p53 and Suv39h1 are mutated or missing. These data further underline the importance of residual dormant senescent cells persistently residing in the tumor after chemotherapy since some of these postsenescent cells bear the risk of undergoing proliferative recovery and with their acquired stemness features are likely to become the drivers of tumor initiation and/or relapse. Thus, further studies addressing senescence‐associated stemness (SAS) and autocrine and paracrine effects of senescent cells along with their SASP are needed to understand and modulate distinct components of the senescent state with the aim of creating a beneficial and therapeutically desirable response.

In this study, we investigated senescence‐associated stemness and its related implications in EpCAM+/CD133+ liver cancer stem cell (LCSC) and EpCAM−/CD133− nonstem cell populations in HuH7 cell line. We demonstrated that treatment with doxorubicin (Dox) induces senescence in both cell populations accompanied with an increase in the expression of stem cell‐related genes. Importantly, Dox treatment significantly increased EpCAM + stem cell population in EpCAM−/CD133− nonstem cells suggesting senescence‐associated reprogramming of nonstem cell population into LCSCs. In addition, when Wnt/β‐catenin signaling was inhibited using Axin2 stabilizer IWR‐1, the number of senescent cells did not change but the senescent‐associated stemness was decreased. Moreover, Dox‐treated EpCAM−/CD133− nonstem cells with acquired stem cell properties had increased *in vivo* tumor‐forming ability when implanted in NSG mice. In addition, when SASP‐conditioned media collected from EpCAM−/CD133− nonstem cells or EpCAM+/CD133+ LCSCs treated with Dox were applied onto hiPSC‐derived hepatocytes, senescence was induced in these mature hepatocytes along with an increased expression of stemness‐related genes *KLF4* and *Axin2*. Taken together, our findings demonstrate therapy‐induced senescence accompanied with an induction of senescence‐associated stemness in EpCAM+/CD133+ LCSC and EpCAM−/CD133− nonstem cell populations. The profound enhancement in tumor‐forming ability of EpCAM−/CD133− nonstem cells undergoing TIS further emphasizes the importance of reevaluating the effect of prosenescence therapy in HCC and supports the potential use of senolytic drugs in HCC treatment in order to eliminate adverse effects of therapy‐induced senescent cells.

## 2 Material and methods

### 2.1 Isolation of LCSCs and Nonstem Cells from HuH‐7

The HuH7 cell line was a gift from Dr. Mehmet Ozturk, IBG, Turkey. The authentication of the HuH7 cell line was achieved by DNA profiling at the University of Colorado Cancer Center (UCCC) DNA Sequencing & Analysis Core (CO, USA) using Applied Biosystem’s Identifiler kit (PN4322288). HuH‐7 cells were grown in RPMI Medium 1640 (GIBCO, Life technologies Europe Bleiswijk, The Netherlands, UK) supplemented with 1% nonessential amino acid (GIBCO, Life Technologies, 3 Fountain Drive; Paisley, UK), 2 mmol·L^−1^ GlutaMAX (GIBCO, Life Technologies) 1% penicillin/streptomycin (GIBCO, Life Technologies, Grand Island, USA), and % 2–10 FBS (GIBCO, Life Technologies; Cat #10500‐064, E.U. Approved [South American]) at 37 °C, %5 CO_2_. The subsets of LCSCs and nonstem cells were enriched from HuH‐7 cells via fluorescence‐activated cell sorting (FACS) as previously described [19].

### 2.2 Doxorubicin treatment

About 7× 10^4^ cells were seeded into 6‐well plates, and after overnight incubation, cells were exposed to 100 nm doxorubicin (Dox) (AppliChem; Cat #A4361, Darmstadt, Germany) for 48 h. At day 2, Dox was replaced with fresh media and cells were followed until day 15 with regular media changes every 4 days. For SASP‐CM experiments, conditioned media were collected at days 6 and 8 after Dox treatment. The SASP‐conditioned media (SASP‐CM) were applied onto hİPSC‐derived hepatocytes after mixing SASP with fresh medium at a 1 : 1 ratio.

### 2.3 Senescence‐associated β‐galactosidase (SA‐βGAL) assay

SA‐βGAL activity was evaluated in the LCSCs and nonstem cells after 2, 4, 6, and 15 days of culture by using the Senescence Detection Kit (BioVision; Cat #K320‐250, Milpitas, USA) per manufacturer's instructions. Briefly, cells were seeded into triplicates in 6‐well plates, washed twice with 1X PBS, and then fixed with formalin for 10 min at room temperature. Next, cells were washed twice with 1 X PBS and incubated overnight at 37 °C with β‐gal substrate in an acidic buffer (pH 6.0). Next day, cells were examined with an inverted microscope and photographed (Olympus BX41). The development of a perinuclear blue color was taken as an indicative of a senescent cell.

### 2.4 Cell sorting of senescent and proliferating cells

The separation of senescent and proliferating cells in the Dox‐treated cultures was done using the combination of cell size and autofluorescence (due to lipofuscin content, measured in FL‐1) as sorting parameters. Sorted cells were then confirmed in cell culture by using other markers of senescent cells including morphology and SA‐β‐gal reactivity [20, 21]. Briefly, cells after Dox treatment were trypsinized and collected in RPMI‐1640 supplemented with 10 % FCS and immediately used for analysis and sorting via a FACS sorter. Autofluorescence of unfixed cells was measured in FL1 (logarithmic). Cell size was monitored by FSC in linear scale. The population of live cells was defined in a FSC/SSC dot plot. After apoptotic cells and debris were gated out, sorting gates were set in a FSC/FL1 dot plot. The upper and lower quartiles with respect to both FSC and FL1 were then used to sort senescent and young proliferating cells, respectively. Cells were sorted at a rate of 100 cells/s.

### 2.5 Quantitative reverse transcription PCR (RT–qPCR)

Total RNA was isolated using the GeneJET RNA purification kit (Thermo Fisher Scientific Baltics UAB; Cat #K0732, Vilnius, Lithuania) according to manufacturer's instructions, and the RNA concentration was detected using NanoDrop (Thermo Fisher Scientific, Serial No:G118, USA). One microgram of RNA was then converted to cDNA using a Maxima First Strand cDNA Synthesis Kit (Thermo Fisher Scientific; Cat #K1642, Vilnius, Lithuania). For RT–qPCR experiments, expression levels were determined in quadruplicate on a 7500 Fast RT PCR System (Applied Biosystems, Life Technologies Holdings Pte Ltd., Singapore), using the TaqMan Universal Master Mix (Thermo Fisher Scientific; Cat #4304437). The relative gene expression was normalized to the *RPL41* gene and calculated by using the 2^−ΔΔCt^ method. The primers are given in Table S1.

### 2.6 Apoptosis assay

Cells were stained with Annexin V and 7‐AAD using FITC Annexin V Apoptosis Detection Kit (BioLegend; Cat #640922, San Diego, USA) as described in manufacturer's instructions. Apoptotic rates were assessed with BD LSR Fortessa using Flowjo software (Becton Dickinson, Heidelberg, Germany).

### 2.7 Cell proliferation assay (EdU staining)

EdU staining was conducted using Click‐iT™ EdU imaging kit (Invitrogen, Carlsbad, CA, USA) according to the manufacturer’s protocol. This protocol was adapted for histological staining of cells grown on the slides. 2 × 10^4^ cells were cultured on glass coverslips and incubated with EdU reagent for 2 h. After removal of EdU containing media, cells were fixed with 4% paraformaldehyde for 15 min and washed twice with 3% bovine serum albumin (BSA) in PBS. The slides were permeabilized with 0.5% Triton X‐100 in PBS for 20 min. After washing twice with 3% BSA in PBS, the slides were incubated with a Click‐iT™ reaction cocktail containing Click‐iT™ reaction buffer, CuSO_4_, Alexa Fluor® 594 Azide, and reaction buffer additive for 30 min while protected from light. The slides were washed once more with 3% BSA in PBS. For subsequent DNA staining, sections were washed once with PBS and then incubated with 0.5 μg/mL DAPI for 4 min. The slides were then washed twice with PBS and mounted with ProLong™ Gold Antifade Mountant (Invitrogen). All steps were carried out at room temperature. For imaging of slides, Confocal LSM 800 (JENA, Germany) was used and figures were prepared using imagej (is developed by contributions worldwide) software.

### 2.8 Production of hİPSC‐derived hepatocytes

Human‐inducible pluripotent stem cells (hiPSCs) were cultured with mTESR1 medium in a Matrigel‐coated culture dish. After the cell density reached 70%, the hİPSC medium was replaced with Roswell Park Memorial Institute / B27 medium containing 100 ng/mL Activin A (PeproTech), 50 ng/mL Wnt3a(R&D Systems), and 10 ng/mL HGF(PeproTech) for 3–5 days to induce endoderm cells. Subsequently, endodermal cells were differentiated to progenitor cells by changing RPMI medium with the knockout [KO]/DMEM containing 20% knockout serum replacement, 1 mm l‐glutamine, 1% nonessential amino acid, 0.1 mm 2‐mercaptoethanol, and 1% dimethyl sulfoxide for further 4 days. In the final stage, cells were cultured with Iscove’s modified Dulbecco's (IMDM) medium containing 20 ng/mL oncostatin M (Invitrogen), 0.5 lM dexamethasone, and 50 mg/mL ITS premix(BD Biosciences) for 5 days in order to obtain mature hepatocytes [22, 23]

### 2.9 Fluorescence staining

For staining, 2 x10^4^ senescent and nonsenescent cells/well were seeded on glass coverslips in 48‐well plates. Next day, cells were fixed by 4% PFA for 15 min and washed twice with 1x PBS, followed by permeabilization with 0.5% Triton X‐100 (Thermo Fisher Scientific, Rockfold, USA) for 10 min. After two more washes with 1x PBS, cells were incubated with the blocking solution containing %10 goat serum (Merck, Steinheim, Germany), % 1 BSA (Merck), %0,001 Tween 20 (Merck), and 0.3 mm Glycine (Merck, Steinheim, Germany) for 1 h at room temperature. After blocking, cells were initially incubated with EpCAM antibody (Miltenyi Biotec; Cat #130‐110‐998, Gladbach, Germany) (1 : 300) for 3h in RT, followed by 3 washes with 1x PBS, and then incubated with secondary antibody (1 : 1000) for 1 h. After the secondary antibody (Abcam; Cat #ab150113, Carlsbad, USA), cells were washed three more times with 1x PBS, then stained with DAPI (0.5 µg/ml) for 4 min and immediately mounted using ProLong™ Gold Antifade Mountant (Invitrogen, Eugene, OR, USA). Cells were visualized using the Confocal LSM 800 microscope.

### 2.10 *In vivo* tumor xenograft assays

All experiments using research animals were performed according to the ethical approval provided by the Animal Ethics Board of Izmir Biomedicine and Genome Center, Turkey (#38/2019). Tumor xenograft studies were performed using untreated and Dox‐treated EpCAM−/CD133− nonstem cells. After two days of Dox treatment (100 nm), cells were incubated with fresh media and collected at day 6 in FBS‐free RPMI‐1640 medium. NSG (NOD‐scid IL2Rgamma^null^) mice were inoculated subcutaneously in the right and left flank region with 1 × 10^4^ cells/200 µL control nonstem cells (left and right flank for 5 mice) and Dox‐treated nonstem cells (left and right flank for 5 mice). The incidence of tumors was assessed semiweekly for first 10 days, then biweekly until mice were sacrificed at day 41 when several tumor volumes reach to ˜ 2mm (tumor volume = (length × (width × width))/2, where length represents the largest tumor diameter and width represents the perpendicular tumor diameter).

### 2.11 TGF‐β reporter assay

Hep3B and Hep3B‐TR cells were seeded at a density of 8 × 10^4^ cell/well in 24‐well plates, and transfection experiments were performed in triplicates using Lipofectamine 2000 according to manufacturer’s protocols. Transfection was done with 200 ng of luciferase reporter plasmid containing four tandem Smad‐binding elements (pSBE4‐Luc; Addgene, Cambridge, MA, USA). In each well, cells were also cotransfected with a 30 ng of pRL‐TK control plasmid (Promega, Madison, WI, USA), encoding Renilla luciferase gene for normalizing transfection efficiency. After 24 h of incubation, transfected cells were treated with 5 ng/mL TGF‐β1 (Sigma) for another 24h. The luciferase reporter assay was performed using Dual‐Glo Luciferase Assay Kit (Promega) according to manufacturer’s protocols. The luminescence intensity of Firefly luciferase was normalized to Renilla luciferase, and results were analyzed using GraphPad prism software.

### 2.12 ELISA

ELISA experiments were performed using Human Common Chemokines Multi‐Analyte ELISArray Kit (Qiagen MEH‐009A) and Human Inflammatory Cytokines Multi‐Analyte ELISArray™ Kit (Qiagen MEH‐004A), respectively, according to manufacturer protocol. Conditioned media were collected from EpCAM−/CD133− control and 100 nm Dox‐treated EpCAM−/CD133− cells on day 6. Briefly, after centrifugation at 1200 rpm for 5 min, 50 µl of conditioned media was added to each well of the array. Results were normalized with negative and positive controls supplied by the kit. Well absorbance was measured at 450 nm as well as at 570 nm to normalize for optical imperfections using a microplate reader (Thermo Scientific Multiskan Go, USA).

### 2.13 Statistics

Statistical analyses were performed using graphpad prism 7 (GraphPad Software, Inc, California, USA) software. Each experiment was repeated at least 3 times independently. All values and error bars were represented as mean ± SD. Two‐tailed unpaired Student’s *t*‐test was used to determine statistical significance between 2 experimental groups. Differences between groups were considered as ‘>0.05’(n.s.), ‘≤0.05’(*), ‘≤0.01’(**), ‘≤0.001’(***), ‘≤ 0.0001’(****).

## 3 Results

### 3.1 **Doxorubicin induces senescence in HCC cell lines and in sorted EpCAM+/CD133+ liver cancer stem cell (LCSC) and EpCAM** −**/CD133**−**nonstem cell populations**

**Fig. 1:**
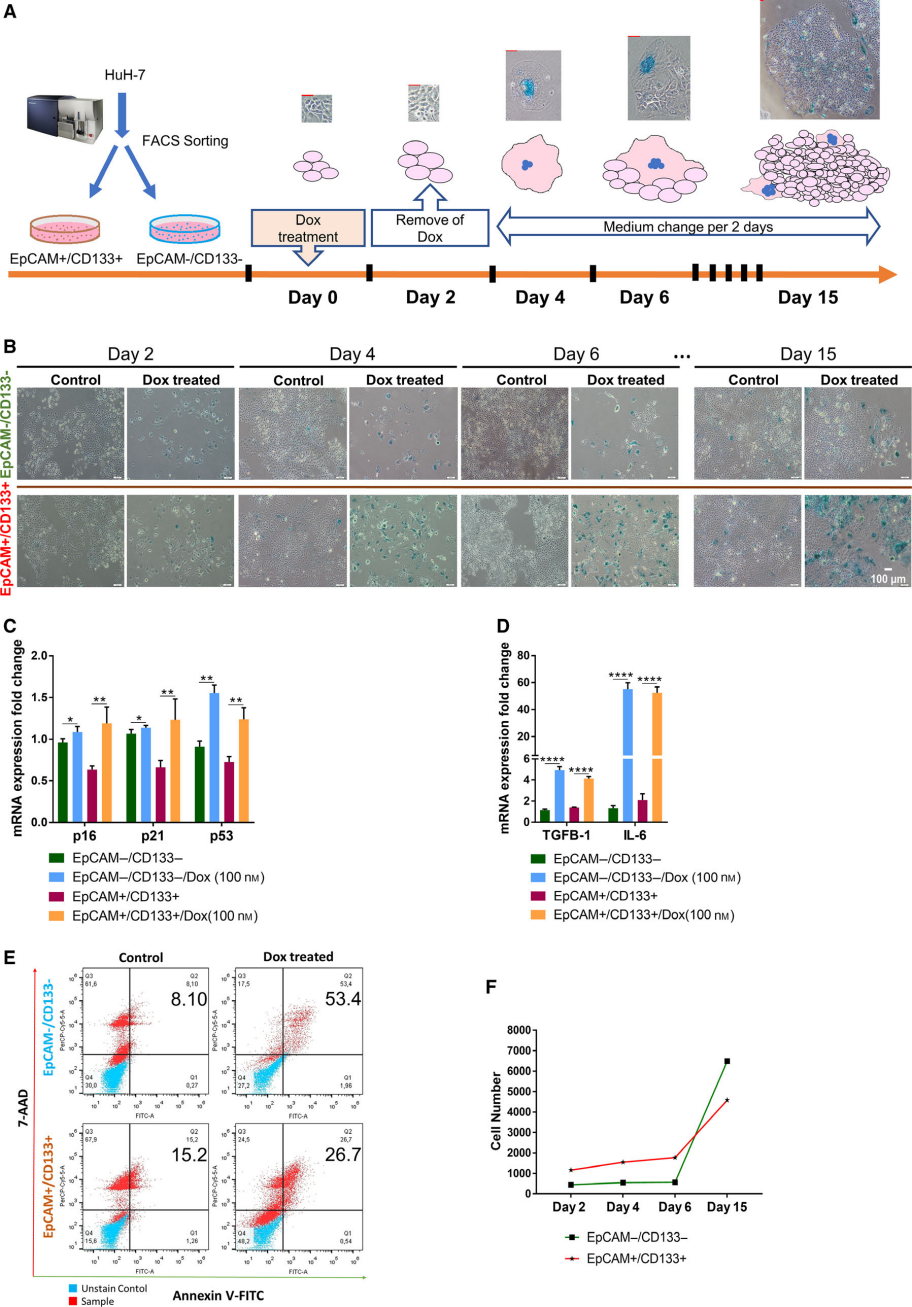
Analysis of induction of senescence and apoptosis in EpCAM+/CD133+ LCSCs and EpCAM‐/CD133‐ nonstem cells after Dox treatment. (A) The experimental workflow of therapy‐induced senescence model. (Scale bars: 50 μm) (B) Light microscope images of Dox‐treated cells after SA‐β‐gal staining on different days. (Scale bars: 100 μm) Expressional analysis of (C) senescence‐associated genes *p16*, *p21*, *p53*, and (D) SASP factors *TGF‐β* and *IL‐6* by qPCR. (E) Flow cytometry analysis of Dox‐induced apoptosis using 7‐AAD/Annexin V staining. (F) Total cell number was counted and graphed for EpCAM+/CD133+ LCSCs and EpCAM−/CD133− nonstem cells on different days after Dox treatment. Two‐tailed unpaired Student’s *t*‐test was used to determine statistical significance. Data represent the average of at least three (n:5) independent experiments. *P* > 0.05 (n.s.), *P* ≤ 0.05 (*), *P* ≤ 0.01(**), *P* ≤ 0.001(***), *P* ≤ 0.0001(****). Error bars indicate standard deviation (SD).

We used doxorubicin to model therapy‐induced senescence in HCC cell lines [24, 25]. Upon Dox treatment, p53 mutant (PLC/PRF/5, SNU449, SNU475) or null (Hep3B) cell lines exhibited low levels of senescence whereas HuH7 cell line carrying mutant p53 gene which results in higher levels of nuclear p53 protein [26, 27] and HepG2 cell line with wt p53 protein displayed higher levels of Dox‐induced senescence. It is noteworthy that even Hep3B cells with null p53 exhibited substantial amount of Dox‐induced senescence, which is consistent with studies indicating only partial p53 dependence in drug‐induced senescence (Fig. S1) [28, 29, 30, 31]. We continued our studies with HuH7 cells from which EpCAM+/CD133+ LCSCs and EpCAM‐/CD133‐ nonstem cells can be isolated via flow cytometry as previously described (Karagonlar et al). After EpCAM+/CD133+ LCSCs and EpCAM−/CD133− nonstem cells were isolated from HuH7 cell line via flow cytometry, these cell populations were treated with 100 nm Dox for 2 days, and then, Dox was replaced with fresh media and cells were followed until day 15 (Fig. 1A). At day 2, only a few cells exhibited flattened and enlarged morphology resembling senescence phenotype; however, at day 6 the majority of the cells showed senescence morphology which was also confirmed by SA‐β‐gal staining (Fig. 1B). We investigated the expression of senescence‐related genes at day 6 in both Dox‐treated LCSC and nonstem cell populations and demonstrated the upregulation of *p16*, *p21*, and *p53* which are known to be upregulated to mediate cell cycle arrest in senescent cells (Fig. 1C). We also detected increased expression of *IL‐6* and *TGF‐β1* which are known to be major components of SASP [11, 32], and which are produced and secreted by senescent cells (Fig. 1D). When we compared apoptosis in Dox‐treated LCSCs and nonstem cells, the percentage of apoptotic cells in EpCAM−/CD133− nonstem cells were significantly higher than the percentage of apoptotic cells in EpCAM+/CD133+ LCSCs (Fig. 1E), consistent with higher drug resistance of the LCSC population. Accordingly, after 2 days of Dox treatment, EpCAM−/CD133− nonstem cell number significantly dropped due to excessive apoptotic death. Cell numbers remained essentially unchanged until day 6 for both populations, suggesting that drug‐treated cells entered growth arrest. After day 6, gradual increase in cell number was observed for both populations. At day 15, total cell number of EpCAM−/CD133− nonstem cells exceeded the total cell number of EpCAM+/CD133+ LCSCs suggesting a faster growth rate for nonstem cells.

### 3.2 **Doxorubicin‐induced senescence contributes to stemness phenotype in both EpCAM+/CD133+ LCSC and EpCAM** −**/CD133**−**nonstem cell populations**

**Fig. 2:**
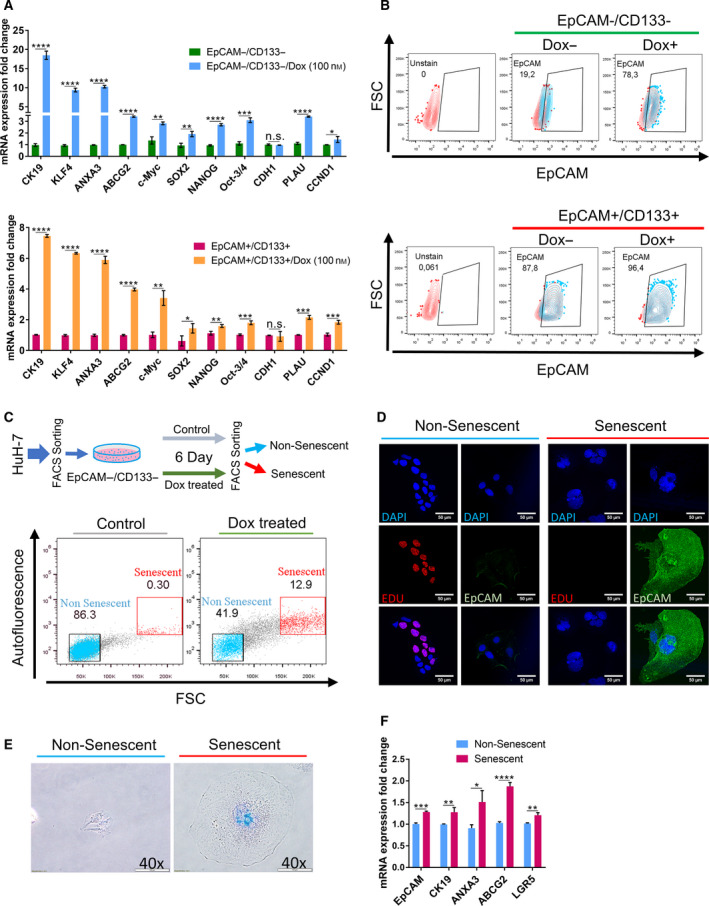
Senescence‐associated stemness was analyzed in EpCAM+/CD133+ LCSCs and EpCAM−/CD133− nonstem cells. (A) Stemness‐related gene expression was analyzed by qPCR in EpCAM+/CD133+ LCSC and EpCAM−/CD133− nonstem cell subpopulations after Dox treatment at day 6. (B) Effect of Dox treatment on EpCAM + cell population was analyzed by flow cytometry using EpCAM‐FITC antibody. (C) Separation of nonsenescent and senescent cells from Dox‐treated EpCAM−/CD133− nonstem cells using size and autofluorescence differences via FACS. Enlarged cells with increased autofluorescence were isolated as senescent cells. (D) Immunofluorescence staining of isolated cells. The isolated senescent cells were negative for EdU staining while these senescent cells had high EpCAM staining. The nonsenescent cells were stained positively for EdU, however, did not show EpCAM expression. (Scale bars: 50 μm) (E) The validation of senescent and nonsenescent cells via SA‐βGal staining. (Scale bars: 100 μm) (F) qPCR analysis showed that senescent cells have increased expression of stemness‐related genes. Two‐tailed unpaired Student’s *t*‐test was used to determine statistical significance. Data represent the average of at least three (n:3) independent experiments. *P* > 0.05(n.s.), *P* ≤ 0.05 (*), *P* ≤ 0.01(**), *P* ≤ 0.001(***), *P* ≤ 0.0001(****). Error bars indicate standard deviation (SD).

In order to understand the effect of Dox‐induced senescence on the stemness phenotype of LCSC and nonstem cell populations, we analyzed the expression of reprogramming and stem cell‐related genes by qPCR. We demonstrated that Dox treatment causes significant increase in the expression of reprogramming genes *SOX2*, *KLF4,* and *c‐MYC* as well as liver stemness‐related genes *EpCAM*, *CK19,* and *ANXA3* and the multidrug resistance‐related gene *ABCG2* in EpCAM−/CD133− nonstem cells (Fig. 2A). Although EpCAM+/CD133+ LCSCs already express higher basal levels of these stem cell‐related genes, Dox treatment was able to even further increase their expressions in the LCSC population. Although EpCAM−/CD133− cells do not express stemness‐related genes in normal culture conditions, we detected a robust increase in the expression of many stemness‐related genes after Dox treatment (Fig. 2A). The expression levels of reprogramming genes *NANOG*, *OCT4,* and *KLF4* even reached to higher levels in nonstem cells compared with LCSCs after Dox treatment (Fig. S2). Moreover, flow cytometry analysis of stem cell surface markers commonly used for isolating liver cancer stem cells demonstrated that, while expression of CD44 and CD90 remained unchanged upon Dox treatment, EpCAM + cell population significantly increased in both Dox‐treated LCSCs and nonstem cells (Fig. S3, Fig 2B). In the nonstem cell population, the percentage of EpCAM + population increased from 19.2% to 78.3% upon Dox treatment (Fig. 2B). In order to understand whether EpCAM expressing cells are actually among the Dox‐induced senescent cell population, we first isolated non‐senescent (or lower senescent) and senescent cells via autofluorescence based cell sorting from Dox‐treated EpCAM−/CD133− nonstem cells as previously described [33]. Nonsenescent and senescent cells were cultured separately after sorting and characterized by SA‐β‐gal reactivity and 5‐ethynyl‐20‐deoxyuridine (EdU) incorporation indicating proliferation via DNA synthesis. We confirmed that the enlarged cells with increased autofluorescence were senescent cells while the cell population sorted out as nonsenescent were EdU‐positive cells still proliferating after Dox treatment (Fig. 2C–E). More importantly, these senescent cells had increased EpCAM staining confirming senescence‐associated induction of EpCAM expression (Fig. 2D,F). In addition to EpCAM expression, compared to nonsenescent counterparts, these senescent cells also had significantly higher expression of stemness‐related genes *CK19*, *ANXA3*, *LGR5,* and *ABCG2* (Fig. 2F).

### 3.3 Inhibition of Wnt/β‐catenin pathway by IWR‐1 decreases expression of stemness markers

**Fig. 3:**
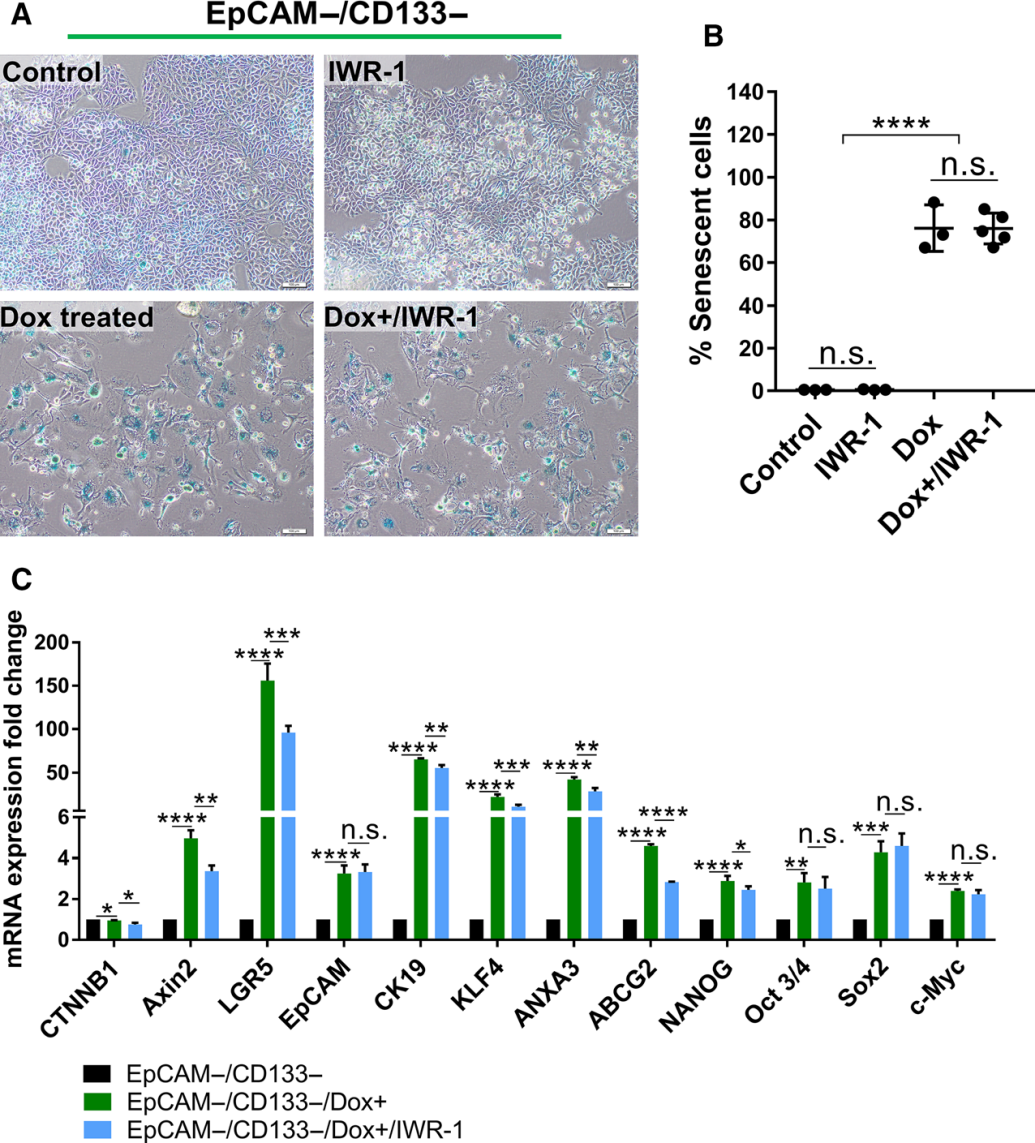
The targeting of canonical Wnt/β‐catenin pathway reduces the expression of stemness‐related genes in EpCAM−/CD133− nonstem cells. (A) SA‐β‐gal staining of untreated and Dox‐treated EpCAM−/CD133− nonstem cells with and without IWR‐1 treatment. (Scale bars: 100 μm) (B) The number of senescent cells was graphed as percentage of the total cell number in all experimental groups. (C) The change in the expression of stemness‐related genes was analyzed by qPCR in all experimental groups. Two‐tailed unpaired Student’s t‐test was used to determine statistical significance. Data represent the average of at least three (n:3) independent experiments. *P* > 0.05 (n.s.), *P* ≤ 0.05 (*), *P* ≤ 0.01 (**), *P* ≤ 0.001 (***), *P* ≤ 0.0001 (****). Error bars indicate standard deviation (SD).

Additionally, we detected a significant increase in the expression of Wnt/β‐catenin target genes *CTNNB1*, *AXIN2*, *PLAU*, *CCDN1,* and *LGR5* suggesting the activation of this signaling pathway by Dox treatment in both LCSC and nonstem cell populations (Fig. 2A). To identify whether Dox‐induced senescence‐associated stemness of the EpCAM−/CD133− nonstem cells depends on Wnt/β‐catenin pathway, we inhibited Wnt/β‐catenin signaling using IWR‐1 which stabilizes the destruction complex member Axin2. Upon inhibition of the Wnt/β‐catenin pathway, cell morphology (Fig. 3A) and the number of senescent cells did not change (Fig. 3B). However, the inhibition of Wnt/β‐catenin pathway caused a downregulation in the Dox‐induced expression of stemness‐related genes LGR5, CK19, *NANOG*, *KLF4*, *ANXA3,* and *ABCG2* (Fig. 3C). Similarly, our experiments with Dox‐treated LCSCs also demonstrated a decrease in the expression of these stemness‐related genes upon Wnt inhibition (Fig. S4). These results suggest that Wnt/β‐catenin pathway might be involved in the regulation of senescence‐associated stemness upon Dox treatment. However, further studies involving knock‐down experiments of specific Wnt/β‐catenin pathway members are required to reveal a direct role of Wnt/β‐catenin pathway in regulating senescence‐associated stemness in these cell populations.

### 3.4 **Dox‐treated EpCAM** −**/CD133**−**nonstem cells acquire high tumor‐initiating capacity**

**Fig. 4:**
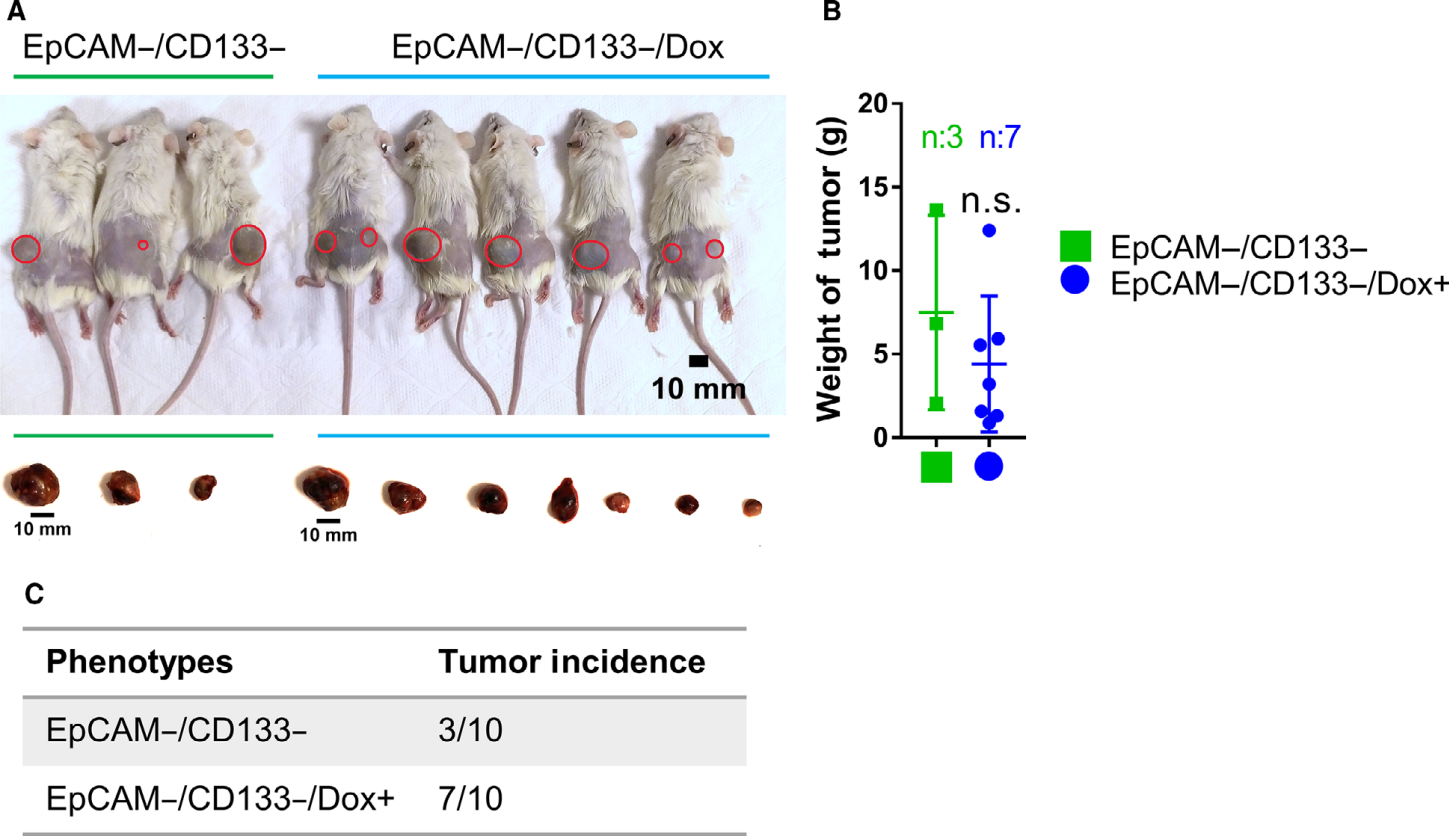
Dox‐treated EpCAM−/CD133− non‐LCSC cells have increased tumorigenicity. (A) NSG mice were subcutaneously injected with 10 000 untreated EpCAM−/CD133− cells or Dox‐treated EpCAM−/CD133− cells and then sacrificed after 41 days. (Scale bars: 10 mm). (B) Tumors were removed from the animals and weighed. (C) Dox‐treated EpCAM−/CD133− nonstem cells had significantly higher tumor incidence than control non‐LCSCs.

EpCAM+/CD133+ LCSCs are characterized by high tumor‐initiating capacity, while EpCAM−/CD133− nonstem cells demonstrate low tumor‐initiating capacity. However, previous research by several groups supports the notion that tumor cells represent a dynamic population with high plasticity, and tumor formation ability of nonstem cells can be modulated by factors that could reprogram these cells into a more dedifferentiated stem cell‐like state [34, 35]. Thus, we tested the tumor behavior of the Dox‐treated nonstem cells with increased stem cell gene expression by subcutaneously injecting these cells into NSG mice. While only 3 of 10 mice injected with untreated EpCAM−/CD133− nonstem cells exhibited a tumor formation, the tumor incidence was 7 out of 10 for the mice injected with Dox‐treated EpCAM−/CD133− nonstem cells. No significant difference was found between the weights of extracted tumors from untreated and treated groups (Fig. 4A–C). These results suggested that Dox treatment renders high *in vivo* tumorigenic ability to low tumorigenic EpCAM−/CD133− nonstem cells.

### 3.5 The conditioned media of Dox‐treated cells contain inflammatory cytokines and induce senescence of hepatocytes in a TGF‐β‐dependent manner

**Fig. 5:**
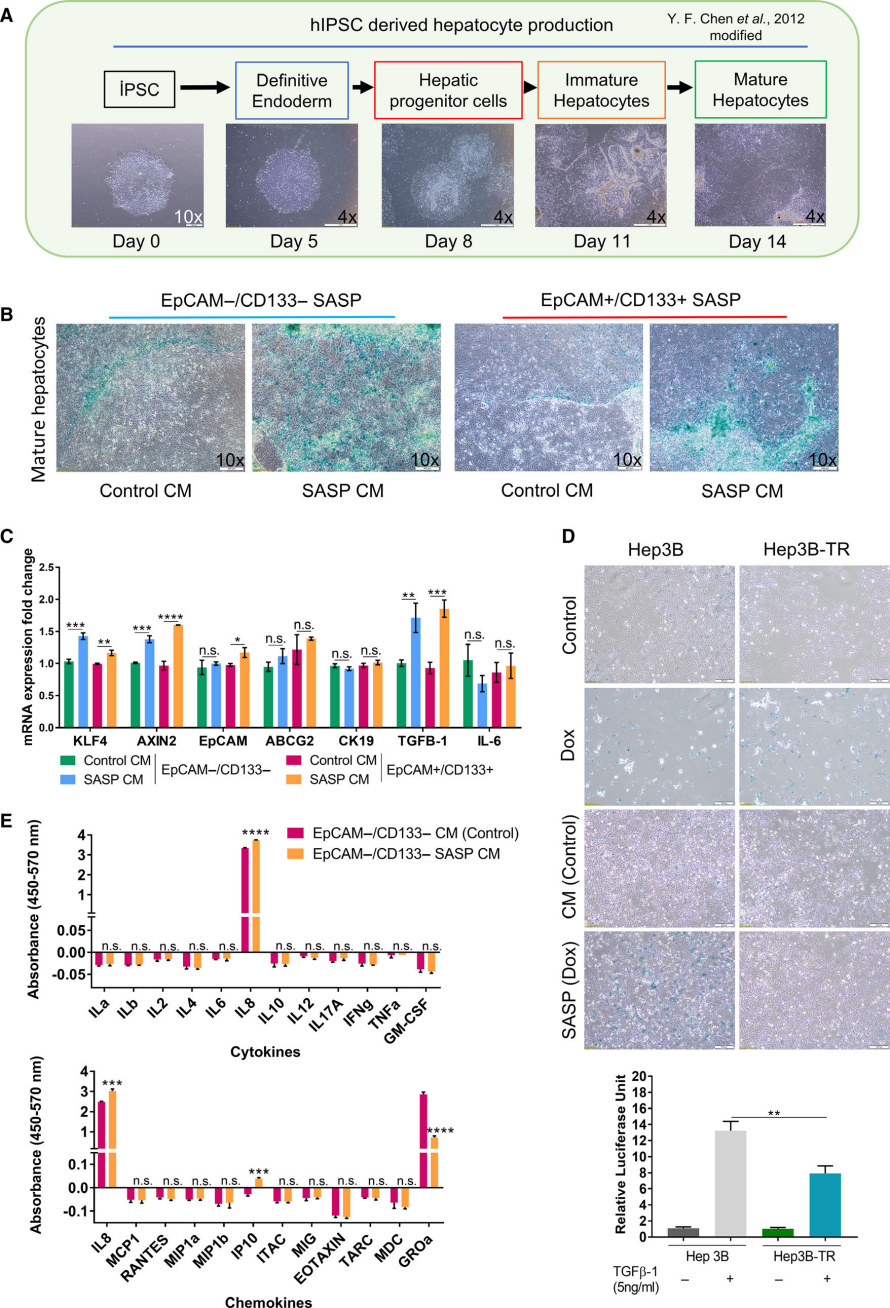
SASP‐CM of Dox‐treated EpCAM+/CD133+ LCSCs and EpCAM−/CD133− nonstem cells induce senescence in hepatocytes. (A) Production of hepatocytes from hiPSCs using our hepatic differentiation protocol. (Scale bars: 200 μm (Day 0) and 1 mm (Days 5–14) (B) The effect of SASP‐CM on hiPSCs derived hepatocytes. Cells were treated with SASP‐CM for 5 days with fresh SASP‐CM added every two days. (Scale bars: 200 μm) (C) The expression of stemness and inflammation‐related genes was analyzed by qPCR. (D) SA‐β‐gal staining of Hep3B and Hep3B‐TR cells incubated with SASP‐CM (top). Luciferase reporter assay using pSBE4‐Luc demonstrated reduced TGF‐β signaling in Hep3B‐TR cells in response to TGF‐β1 treatment (bottom). (Scale bars: 200 μm) (E) SASP‐CM from EpCAM−/CD133− control and 100 nm Dox‐treated EpCAM−/CD133− cells were analyzed using Human Common Chemokines Multi‐Analyte ELISArray Kit (Qiagen MEH‐009A) and Human Inflammatory Cytokines Multi‐Analyte ELISArray Kit (Qiagen MEH‐004A), respectively. Two‐tailed unpaired Student’s *t*‐test was used to determine statistical significance. Data represent the average of at least three (n:3) independent experiments. *P* > 0.05(n.s.), *P* ≤ 0.05 (*), *P* ≤ 0.01(**), *P* ≤ 0.001(***), *P* ≤ 0.0001(****). Error bars indicate standard deviation (SD).

It has been shown that senescent cells can, via their SASP, modulate the tumor microenvironment and either contribute to tumor suppression through recruitment of immune cells or, depending on the context, promote tumor progression. Moreover, transient exposure to the SASP was found to promote cell dedifferentiation and reprogramming [13, 36]. To assess the effect of SASP on hepatocytes, we collected SASP‐condition media (CM) from Dox‐treated EpCAM+/CD133+ LCSCs and EpCAM−/CD133− nonstem cells (Fig. S5). We assessed the effect of SASP‐CM on hepatocytes produced from hİPSC using our previously published hepatic differentiation protocol. These hiPSC‐derived hepatocytes provide a suitable model with many of the characteristics of primary hepatocytes, including lipid storage, albumin secretion, accumulation of glycogen, active uptake of low‐density lipoproteins, and synthesis of urea [22]. Incubating hiPSC‐derived hepatocytes with SASP‐CM from either EpCAM+/CD133+ LCSCs or EpCAM−/CD133− nonstem cells induced senescence in these cells (Fig. 5B). Also, these SASP‐CM‐treated hepatocytes had increased expression of stemness‐related genes *KLF4* and *AXIN2*. Moreover, the expression of *TGF*‐β1 which is an important component of the inflammatory group of molecules secreted by senescent cells and a possible senescence inducer [37] was also significantly increased in these hepatocytes. Importantly, we also analyzed the effect of SASP‐CM on Hep3BTR cells which were established from a human hepatoma line sensitive to TGF‐β (Hep3B‐TS) by exposure to low TGF‐β1 concentrations. These cells show resistance to the inhibitory action of TGF‐β1 on growth due to the loss of the *TGF‐*β*RII* gene upon *in vitro* selection (Hasegawa, 1992; Inagaki, 1993). We also confirmed the reduced TGF‐β signaling in these cells in response to TGF‐β1 treatment using a reporter plasmid consisting of TGF*‐*β responsive Smad‐binding elements (Fig. 5D). Notably, while Dox treatment induced senescence in both Hep3B‐TS and Hep3B‐TR cells, induction of senescence by SASP‐CM incubation was only detected in Hep3B‐TS cells. These results also suggested that TGF‐β signaling pathway activation is required for the induction of senescence through SASP‐CM in a paracrine manner (Fig. 5D). Moreover, we investigated Dox‐induced cytokines and chemokines in EpCAM−/CD133− SASP‐CM using QIAGEN Multi‐Analyte ELISA kits which allowed us to screen for 23 molecules simultaneously. Our data showed that conditioned media of EpCAM−/CD133− nonstem cells contain high amounts of IL8 and GrOα which are well‐known chemotactic inflammatory cytokines associated with senescence [10, 11]. Notably, after Dox treatment there was a further increase for IL8, whereas the amount of GrOα was decreased (Fig. 5E). In addition, the amount of chemokine interferon‐γ‐inducible protein 10 (IP10), which is an important factor in acute‐phase graft injury [38, 39], was increased in SASP‐CM of Dox‐treated EpCAM−/CD133− nonstem cells. These results indicated the basal secretion of inflammatory cytokines IL8 and GrOα by EpCAM−/CD133− nonstem cells and demonstrated that the secretion of IL8 and IP10 can be induced in these cells by Dox treatment.

## 4 Discussion

Cellular senescence has been suggested as a barrier for initiation and progression of HCC [40, 41, 42]. Consistent with the proposed role of cellular senescence as a tumor‐suppressive mechanism, the abrogation of the senescence program due to p53 mutation causes aggressive HCC development [43], while restoration of p53 in liver tumors was shown to trigger immune cell‐mediated tumor clearance [42, 44]. However, more recent studies indicate dual and opposing roles of senescence in HCC and various other cancers.

In the last few years, new research which focused on the role of senescence on cancer stemness and dormancy demonstrated that senescent cells while undergoing senescence arrest paradoxically exhibit increased markers normally associated with stemness. Sabisz and Sklandanowski (2009) determined that about 1% of the nonsmall lung adenocarcinoma A549 cells treated with DNA damaging drugs escaped senescence and lead to regrowth of tumor cell population accompanied with an increase in cancer stem cell marker expression (CD34 and CD117) [45]. Another study by Achutan et al. (2011) involving multiple breast cancer cell lines (MCF‐7, MDA MB231, and T47D) and primary tumors also revealed that cells that escaped from doxorubicin‐induced senescence exhibit stem cell characteristics with increased levels of the stem cell markers CD133 and OCT4 [46]. A more recent report by Milanovic et al. (2018) nicely demonstrated the interplay between senescence and stemness by showing that enhanced stemness detected in adriamycin‐treated cells was absent in adriamycin‐treated cells which failed to undergo senescence due to the absence of Suv39h1, the enzyme responsible for the senescence‐associated epigenetic mark, H3K9Me3. Moreover, using an inducible expression model for p53 and Suv39h1, the authors were able to demonstrate that upon deactivation of these senescence‐essential genes, senescent cells can resume proliferation supporting the idea that senescence can be a reversible condition, when essential senescence maintenance genes are mutated or absent. Importantly, stem cell properties were still conserved in postsenescent cells that escaped TIS and these cells were more aggressive, forming rapidly growing colonies *in vitro* and more malignant tumors when implanted *in vivo* [18, 24].

These observations in the literature point out with experimental evidence that tumor cells carry the potential to re‐enter the cell cycle after senescence. Moreover, since TIS can also be accompanied with reprogramming into a more stem cell‐like state and a more aggressive phenotype, these postsenescent cells might represent an important component of treatment failure and cancer relapse. However, these experiments relating to the effect of TIS on tumor cells have generally been based on studies in bulk culture without considering the different properties of subpopulations in tumor. Tumor heterogeneity at the molecular and genetic levels is a well‐known phenomenon that greatly complicates the diagnosis and treatment of cancer. Accumulating evidence suggests that a small group of cells within a tumor termed cancer stem cells (CSCs) are primarily responsible for this diversity due to their high self‐renewal and differentiation capacities. Adding to this complexity, recent studies have shown that cancer cells can exhibit a high level of plasticity or the ability to dynamically switch between CSC and non‐CSC states [19, 47, 48]. According to the cancer stem cell plasticity model, various factors and/or stimuli within the tumor microenvironment could influence differentiated tumor cells to dedifferentiate and acquire stem cell characteristics. Conversely, cellular factors could also drive CSC differentiation into non‐CSCs. Moreover, studies indicate that *CSC*s exhibit higher *drug resistance* and the generation/selection of chemo‐resistant fraction of CSC after treatment is responsible for tumor regrowth and cancer relapse [49, 50, 51, 52, 53]. Thus, exploration of TIS on cancer stem cells is vital to be able to evaluate the efficacy of prosenescence therapy and/or for planning of following secondary therapeutic interventions.

In our study, we assessed the effect of doxorubicin‐induced senescence on LCSC and nonstem cell population isolated from HuH7 cell line according to their surface markers EpCAM and CD133. EpCAM+/CD133+ LCSCs are characterized by higher differentiation capacity, increased drug resistance and colony‐formation ability, preferential expression of stem cell‐related genes, and stronger tumorigenicity [19, 52]. We also confirmed that our sorted EpCAM−/CD133− or EpCAM+/CD133+ cell populations are not enriched for other common liver cancer stem cell markers CD44 or CD90 (Fig. S3). We detected doxorubicin‐induced senescence in both LCSC and nonstem cell populations while LCSCs showed lower rates of doxorubicin‐induced apoptosis due to their stemness properties [52, 54, 55]. Our gene expression analysis demonstrated that doxorubicin treatment causes upregulation of stemness‐related genes in both populations. Although these stemness‐related genes are already expressed by EpCAM+/CD133+ LCSCs, doxorubicin treatment was able to even further increase their expression levels. On the other hand, EpCAM−/CD133− nonstem cells do not express stemness‐related genes basally; however, we detected a robust increase in the expression of these genes after doxorubicin treatment (Fig. 2A). Moreover, by flow cytometry analysis we detected a significant increase in the percentage of EpCAM + stem cell population in nonstem cells suggesting senescence‐associated reprogramming of nonstem cell population into LCSC population. Moreover, when we sorted out senescent cells from doxorubicin‐treated nonstem cell population using flow cytometry, we were able to demonstrate that the senescent cells rather than the nonsenescent cells among the nonstem cell population exhibit higher levels of EpCAM expression (Fig. 2D), and stemness‐related gene expression (Fig. 2F). Although we cannot completely exclude alternative explanations, our data strongly suggest that senescence is accompanied with an increase in stemness in these cells and nonstem cells undergoing senescence are the ones that acquire stem cell‐like properties. Moreover, it is noteworthy that when we analyzed EpCAM and KLF4 expressions, the cell lines with higher senescence rates due to p53 status (HuH7 and HepG2) also demonstrated higher levels of induction for these stem cell genes, again supporting the relationship between senescence and stemness upon Dox treatment (Fig. S1).

Several studies show that the Wnt/β‐catenin pathway is important for driving self‐renewal of cancer stem cells [56, 57, 58]. Wnt/β‐catenin pathway is also implicated in the control of senescence [18, 24, 59, 60, 61, 62, 63]. In our study, Dox treatment significantly increased Wnt/β‐catenin pathway‐related genes *CTNNB1*, *AXIN2*, *LGR5, CCND1,* and *PLAU* in both LCSCs and nonstem cells. Moreover, when we used IWR‐1, which is known to block Wnt/β‐catenin signaling by stabilizing Axin2 and promoting β‐catenin destruction [64], Dox‐induced expression of several stem cell markers was decreased. However, the inhibition of Wnt/β‐catenin pathway did not significantly alter the number or morphology of senescent cells. Thus, our results suggest a role for Wnt/β‐catenin pathway in the regulation of senescence‐associated stemness upon Dox treatment. However, our results rely on chemical inhibition of Wnt/β‐catenin signaling by IWR‐1. Thus, in order to reveal a direct role of this signaling pathway in senescence‐associated stemness, experiments involving knocking down of specific Wnt/ β‐catenin pathway members hold great importance.

Many studies observed that senescence also may spread to neighboring cells via SASP factors in a paracrine fashion. SASP factors under various conditions induce tumorigenic properties such as chronic inflammation, angiogenesis, drug resistance, and increased metastatic potential [13, 18, 65, 66]. Recently, Eggert et al. (2016) showed that while SASP of senescent hepatocytes suppresses liver cancer initiation by promoting myeloid cell accumulation, SASP was also shown to accelerate the growth of fully established HCC by inhibiting NK cell function through immunosuppressive immature myeloid cell accumulation in murine and human HCC [67]. These studies while further underlying the importance of latent senescence cells and SASP also indicate that senescence might have changing roles during the dynamic and evolving nature of tumors through autocrine or paracrine mechanisms. When SASP‐conditioned media collected from Dox‐treated EpCAM+/CD133+ LCSCs or EpCAM−/CD133− nonstem cells were applied onto hiPSC‐derived hepatocytes, senescence was induced in these mature hepatocytes along with an increased expression of *KLF4*, *AXIN2,* and *TGFβ‐1* demonstrating paracrine effects of Dox‐induced senescent cells on hepatocytes through SASP. Moreover, SASP‐CM did not induce senescence in Hep3BTR cells with reduced TGF‐β signaling, suggesting TGF‐β signaling pathway activation is required for the induction of senescence through SASP‐CM.

Importantly, KLF4 was induced both by Dox treatment and SASP‐CM incubation in senescent cells. Our previous work has demonstrated that KLF4 induces partial reprogramming of EpCAM−/CD133− nonstem cells, accompanied with an increase in stemness and tumorigenicity (Karagonlar *et al*., 2020). However, when we overexpressed KLF4 in various HCC cell lines, we detected neither inhibition nor enhancement of Dox‐induced senescence by KLF4 overexpression (Fig. S6). These data suggest that KLF4 does not have a direct role in the induction of senescence. Nevertheless increased KLF4 expression, we detected in senescent cells still can have important implications in tumorigenesis and therapy response through inducing dedifferentiation of tumor cells. Thus, further experiments would be valuable to evaluate the effect of SASP on dedifferentiation of hepatocytes and the induction of cancer stem cells.

Moreover, our ELISA experiments indicated that there is a basal secretion of inflammatory cytokines IL8 and GrOα by EpCAM−/CD133− nonstem cells. In addition to the enhanced secretion of IL8 upon Dox treatment, the secretion of IP10 was also induced in Dox‐treated cells. Interleukin‐8 (IL‐8) is a known mediator of inflammation and carcinogenesis through its activities related to stem cells, angiogenesis, and metastasis [10, 38, 68]. High IL‐8 levels have been associated with poor prognosis in many malignancies including HCC [10, 68, 69, 70, 71]. Furthermore, induction of IL‐8 by Dox was shown to increase the proportion of stem cells in HCC cell lines, while inhibiting the IL‐8 signaling pathway decreases stem cell population and attenuates drug resistance [68]. Our findings also suggest that IL8 might mediate the bridge between senescence and stemness in HCC and can be an important mediator of the cancer‐promoting properties of senescent tumor cells. In addition, IP10, which was identified as a distinct gene signature of acute‐phase graft injury and late‐phase tumor recurrence after liver transplantation, was induced after Dox treatment. Post‐transplant enhanced IP10 signaling was shown to increase tumor cell proliferation and invasiveness, and promote tumor angiogenesis by mobilizing circulating endothelial progenitor cells into liver graft during liver tumor recurrence after liver transplantation [39]. Further research should aim to investigate the contribution of these cytokines to senescence‐associated stemness and tumorigenicity and determine whether IL‐8 and IP‐10 have a role in the procarcinogenic effect of TIS.

Importantly, similar to the study by Milanovic et al. (2018) we found that the doxorubicin‐treated nonstem cells exhibit higher tumor initiation capacity *in vivo* (Fig. 4). Our data contribute to accumulating evidence pointing to an unexpected tumor‐promoting capability of the senescence program, especially through senescence‐associated stemness and SASP. Wang et al. [72] recently demonstrated that eradication of therapy‐induced senescent cells by a secondary agent can decrease tumor burden and improve survival in xenograft models of human HCC cell lines and in a HCC mouse model. Our data also support the idea that approaches to target senescent cells and their SASP by a secondary therapeutic intervention might be therapeutically important. However, further experiments are required to identify molecular mechanisms mediating the tumorigenicity of therapy‐induced senescence in HCC.

## 5 Conclusion

It is becoming clear that the induction of senescence represents a double‐edged sword for tumor control, and carefully dissecting the beneficial and detrimental aspects of the senescence program is required to harness its therapeutic use. Although the majority of a senescent population is likely to be indefinitely arrested, many recent studies suggest the possibility that TIS could result in the survival of a subpopulation of tumor cells with increased stemness and tumorigenicity. Our study suggests that both LCSC and nonstem cell populations are susceptible to doxorubicin‐induced senescence and senescence‐associated stemness. Thus, targeting senescent cells by senolytic agents which selectively induce removal of senescent cells constitutes a clinically relevant strategy to eliminate adverse effects of therapy‐induced senescent cells and prevent cancer recurrence and relapse.

## Conflicts of interest

The authors declare no conflict of interest.

## Author Contributions

MK and SA contributed to methodology. MK experimented the study. MK, SA, ZFK, and EE wrote—original draft preparation. MK, ZFK, and EE wrote—review and editing. ZFK and EE supervised the study. ZFK and EE involved in funding acquisition. All authors have read and agreed to the published version of the manuscript.

## Supporting information


**Fig. S1.** Dox‐induced senescence in HCC cell lines with different p53 status.

**Fig. S2.** The expression of stem cell markers in untreated and Dox treated EpCAM‐/CD133‐ and EpCAM+/CD133+ cells was determined by qPCR.

**Fig. S3.** The expression of CD44 and CD90 in untreated and Dox treated EpCAM‐/CD133‐ and EpCAM+/CD133+ cells was determined via flow cytometry using CD44‐FITC and CD90‐APC antibodies.

**Fig. S4.** The inhibition of canonical Wnt/β‐catenin pathway reduces the expression of stemness‐ related genes in EpCAM+/CD133+ LCSCs.

**Fig. S5.** SASP‐CM production and collection steps.

**Fig. S6.** The effect of KLF4‐overexpression on Dox‐induced senescence.

**Table S1.** qPCR primers used in the study.
