# Supplementary figures and images for: Doxorubicin‐induced senescence promotes stemness and tumorigenicity in EpCAM−/CD133− nonstem cell population in hepatocellular carcinoma cell line, HuH‐7
Source: Mol Oncol. 2021 Mar 8;15(8):2185–202. doi: 10.1002/1878-0261.12916 (PMC8334288; doi:10.1002/1878-0261.12916)

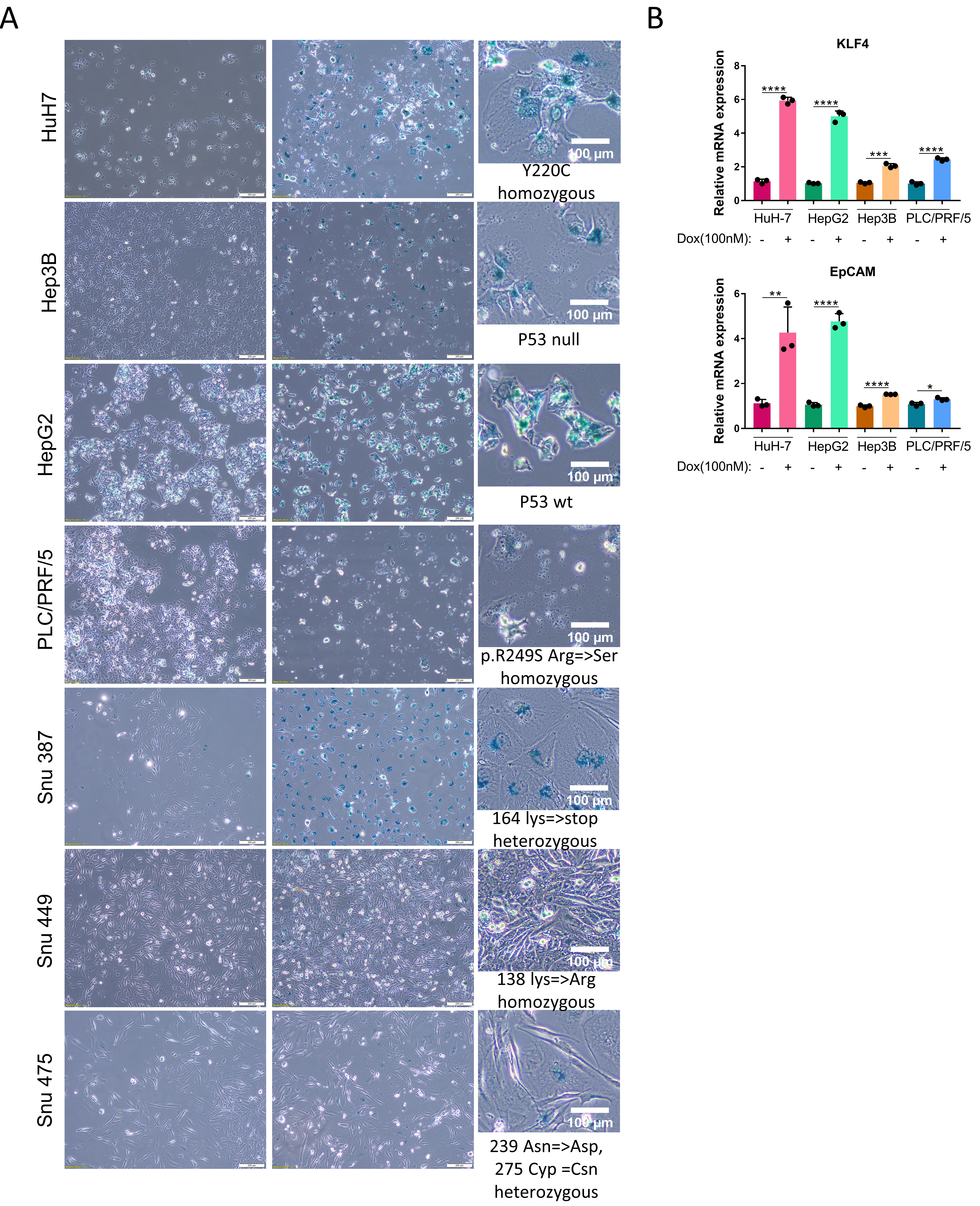

Supplement: Supplementary file 1 — Fig. S1. Dox‐induced senescence in HCC cell lines with different p53 status. [file MOL2-15-2185-s002.tif]

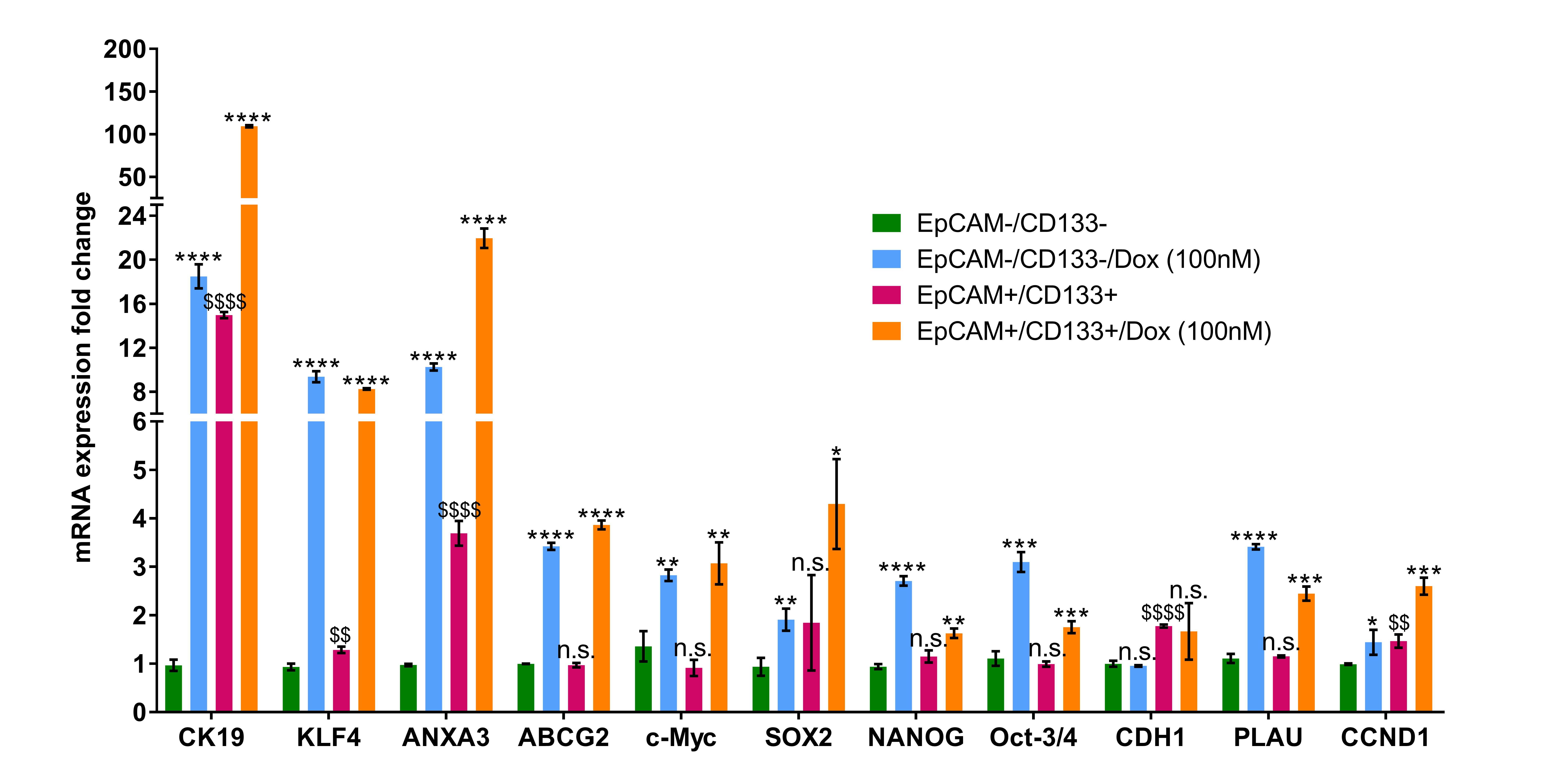

Supplement: Supplementary file 2 — Fig. S2. The expression of stem cell markers in untreated and Dox treated EpCAM‐/CD133‐ and EpCAM+/CD133+ cells was determined by qPCR. [file MOL2-15-2185-s006.tif]

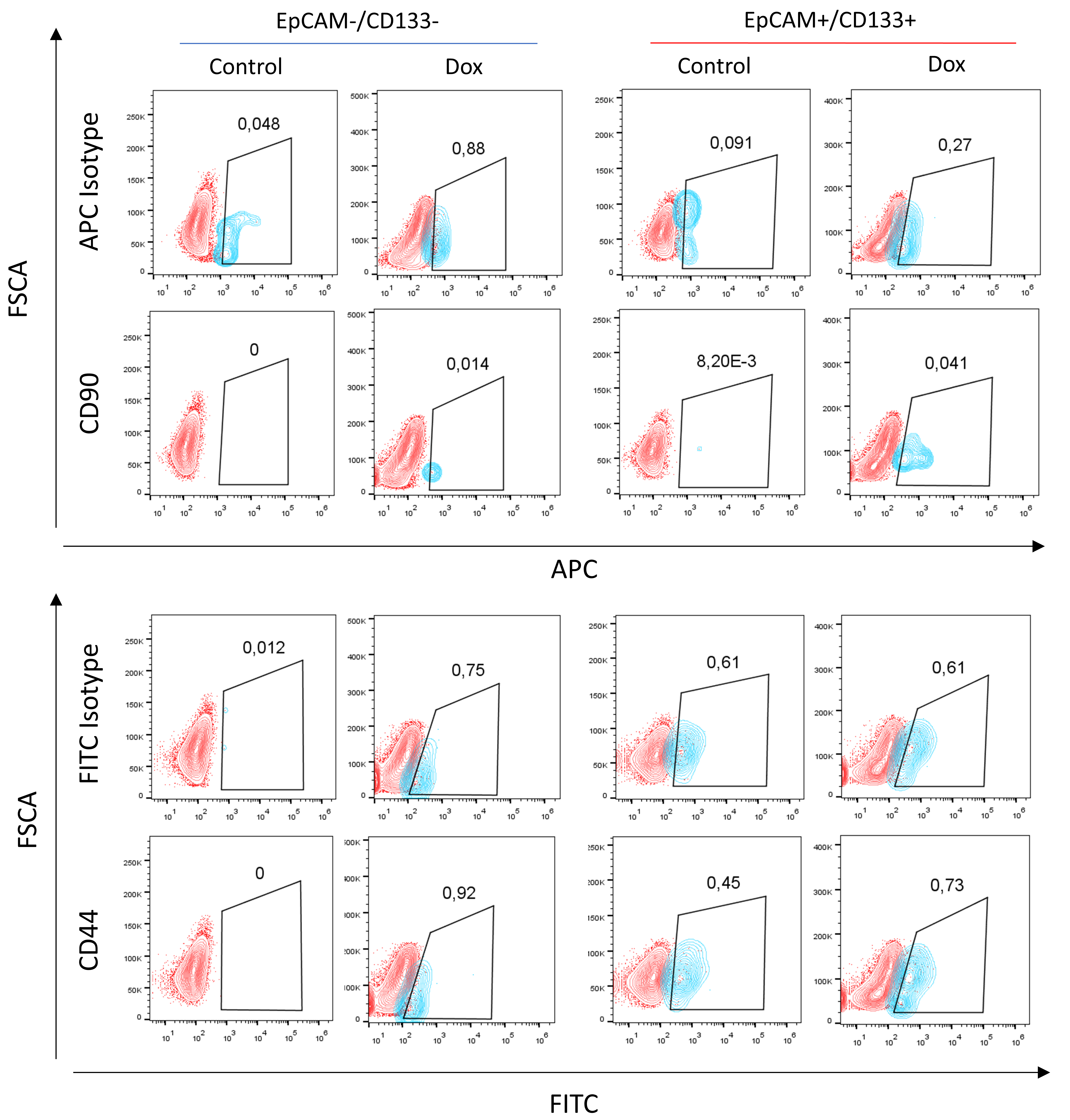

Supplement: Supplementary file 3 — Fig. S3. The expression of CD44 and CD90 in untreated and Dox treated EpCAM‐/CD133‐ and EpCAM+/CD133+ cells was determined via flow cytometry using CD44‐FITC and CD90‐APC antibodies. [file MOL2-15-2185-s007.tif]

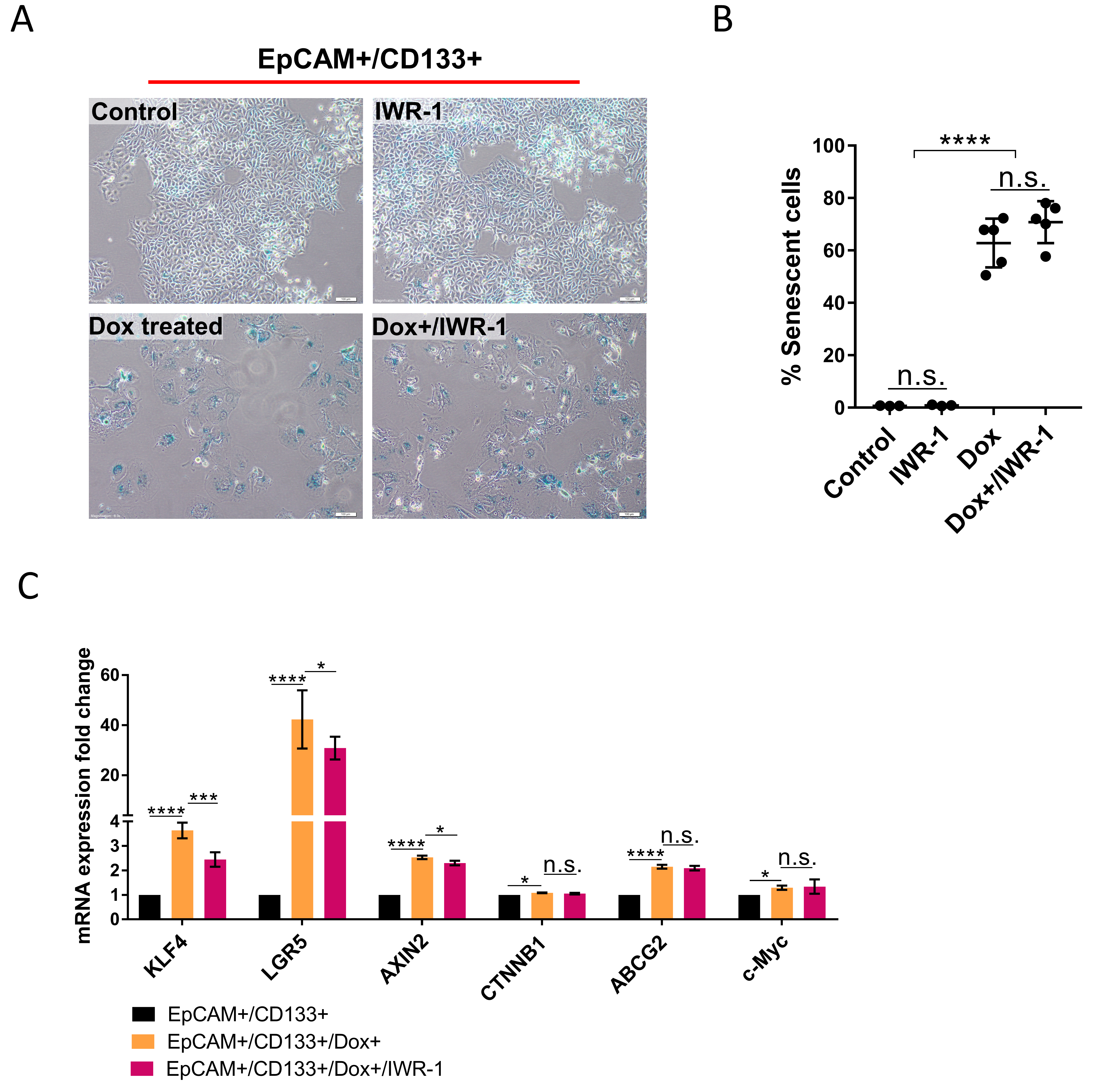

Supplement: Supplementary file 4 — Fig. S4. The inhibition of canonical Wnt/β‐catenin pathway reduces the expression of stemness‐ related genes in EpCAM+/CD133+ LCSCs. [file MOL2-15-2185-s005.tif]

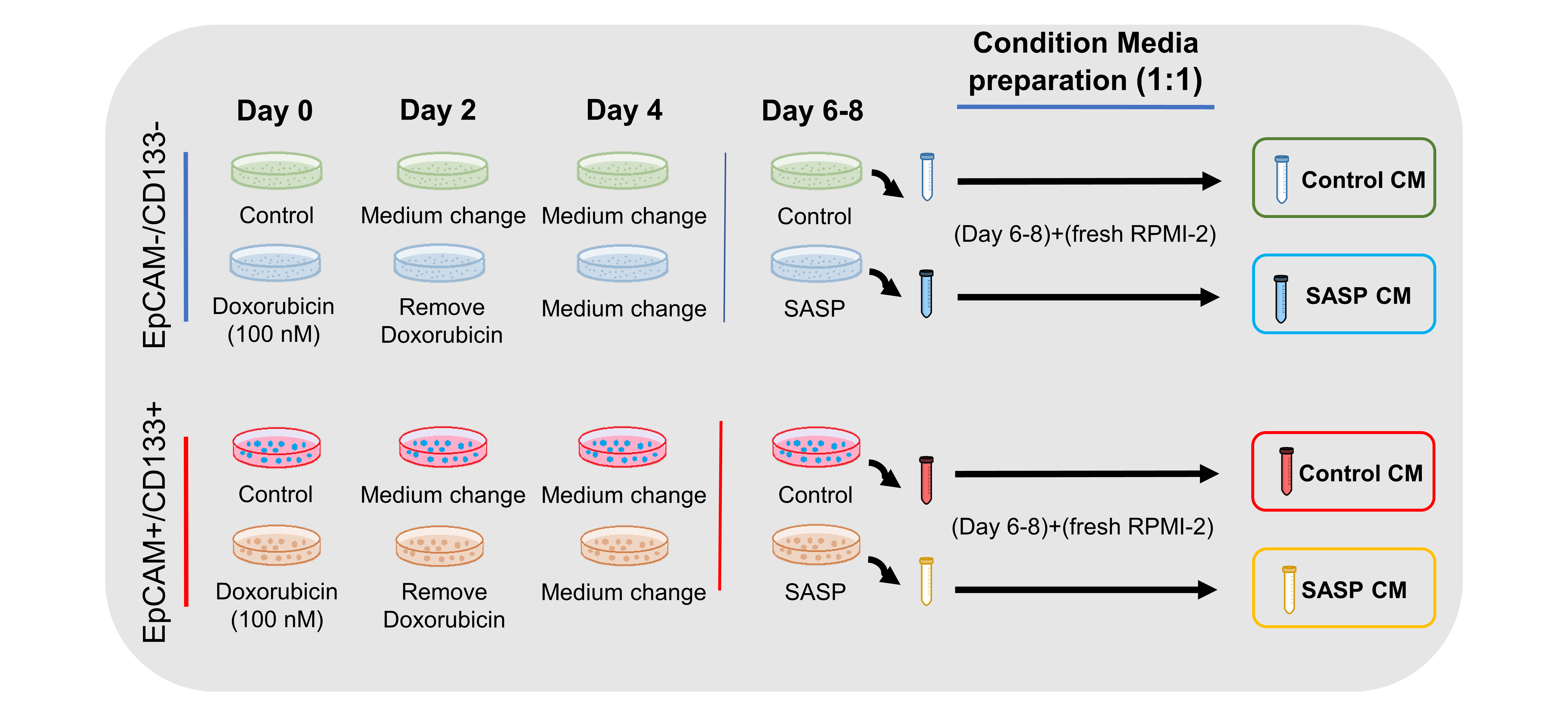

Supplement: Supplementary file 5 — Fig. S5. SASP‐CM production and collection steps. [file MOL2-15-2185-s001.tif]

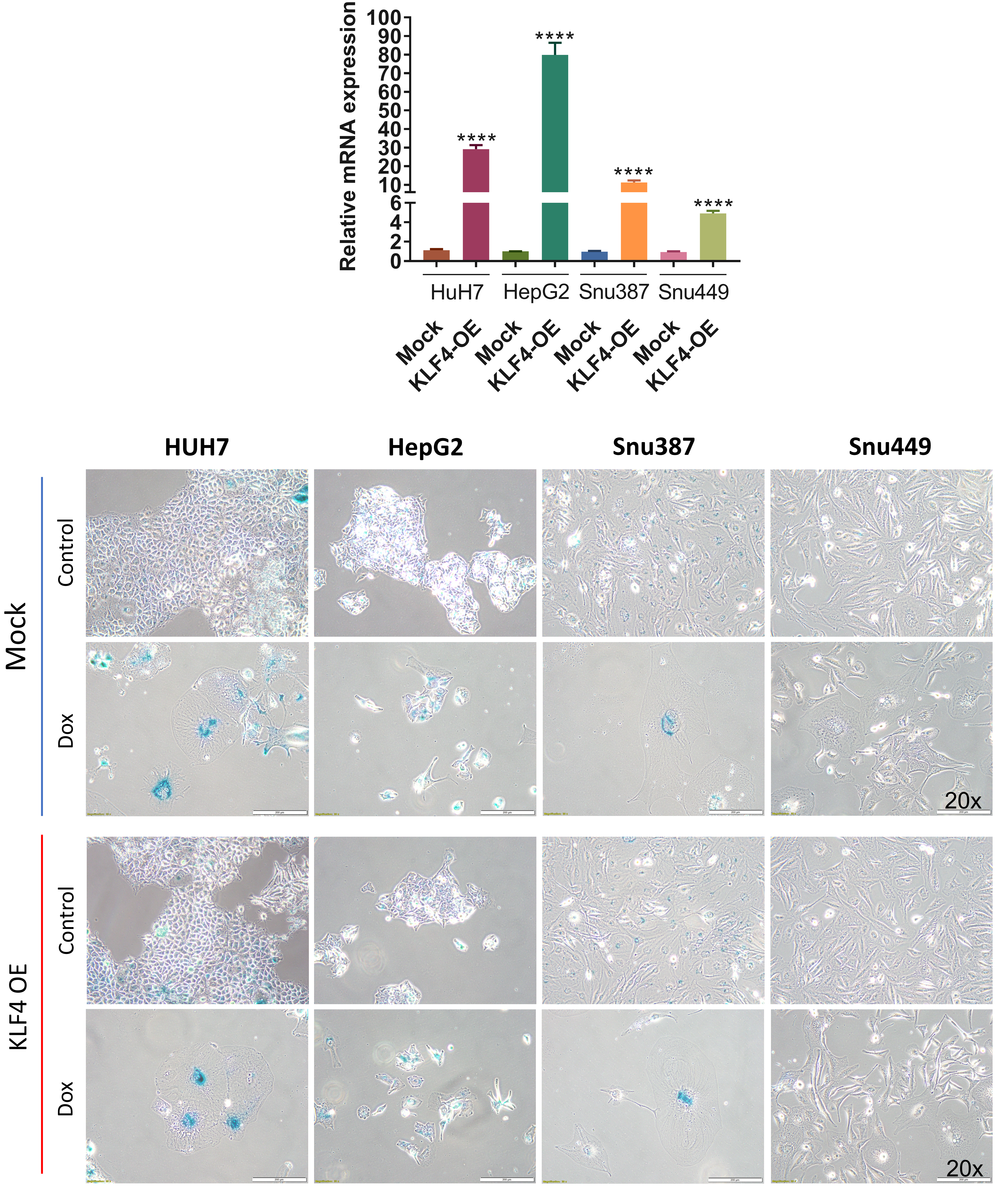

Supplement: Supplementary file 6 — Fig. S6. The effect of KLF4‐overexpression on Dox‐induced senescence. [file MOL2-15-2185-s004.tif]
