# Supplementary material for: Doxorubicin‐induced senescence promotes stemness and tumorigenicity in EpCAM−/CD133− nonstem cell population in hepatocellular carcinoma cell line, HuH‐7
Source: Mol Oncol. 2021 Mar 8;15(8):2185–202. doi: 10.1002/1878-0261.12916 (PMC8334288; doi:10.1002/1878-0261.12916)
Supplement: Supplementary file 7 — Table S1. qPCR primers used in the study. [file MOL2-15-2185-s003.docx]

**Supplementary Table 1: qPCR primers used in the study.**

| **Gene ID** | **Forward primer** | **Reverse primer** | **Prop no** |
| --- | --- | --- | --- |
| **ABCG2** | ttccacgatatggatttacgg | gtttcctgttgcattgagtcc | 29 |
| **ANXA3** | cagaaatatcagccaaaaggacat | ggcgtgttcctcacacaat | 56 |
| **AXIN2** | gatatccagtgatgcgctga | actgcccacacgataaggag | 56 |
| **CCND1** | cggactacaggggagttttg | ctctgctgctcgctgctac | 1 |
| **CK19** | agtaccagcggctcatgg | cttcctgtccctcgagca | 15 |
| **c-Myc** | tgctccatgaggagacacc | cctcatcttcttgttcctcca | 77 |
| **CTNNB1** | tgttaaattcttggctattacgaca | ccaccactagccagtatgatga | 8 |
| **CDH1** | accccctgttggtgtcttta | tgtatgtggcaatgcgttct | 41 |
| **EpCAM** | ccatgtgctggtgtgtgaa | tgtgttttagttcaatgatgatcca | 3 |
| **IL-6** | gatgagtacaaaagtcctgatcca | ctgcagccactggttctgt | 40 |
| **KLF4** | cgttccagtgccaaaaatg | catgtgtaaggcgaggtggt | 85 |
| **LGR5** | accagactatgcctttggaaac | tcccagggagtggattctatt | 78 |
| **NANOG** | tctccaacatcctgaacctca | ttgctattcttcggccagtt | 87 |
| **NOTCH1** | cggggctaacaaagatatgc | caccttggcggtctcgta | 52 |
| **Oct-3/4** | tgaagaacaagtgccaaatagc | ccagcggctatacaaagtgg | 4 |
| **p16** | gagcagcatggagccttc | cgtaactattcggtgcgttg | 20 |
| **p21** | tcactgtcttgtacccttgtgc | ggcgtttggagtggtagaaa | 32 |
| **p53** | ctgagcagccaccctttg | tccttggaggccagacat | 27 |
| **PLAU** | ggggctctgtcacctacg | tcctccttctttgggtaatcaat | 16 |
| **RPL41** | ggccttagcgccattttt | ttggacctctgcctcatctt | 41 |
| **SOX2** | ttgctgcctctttaagactagga | taagcctggggctcaaact | 35 |
| **TGFB-1** | ggagcggaggaaggagtc | ctcttctcccgaccagctc | 63 |


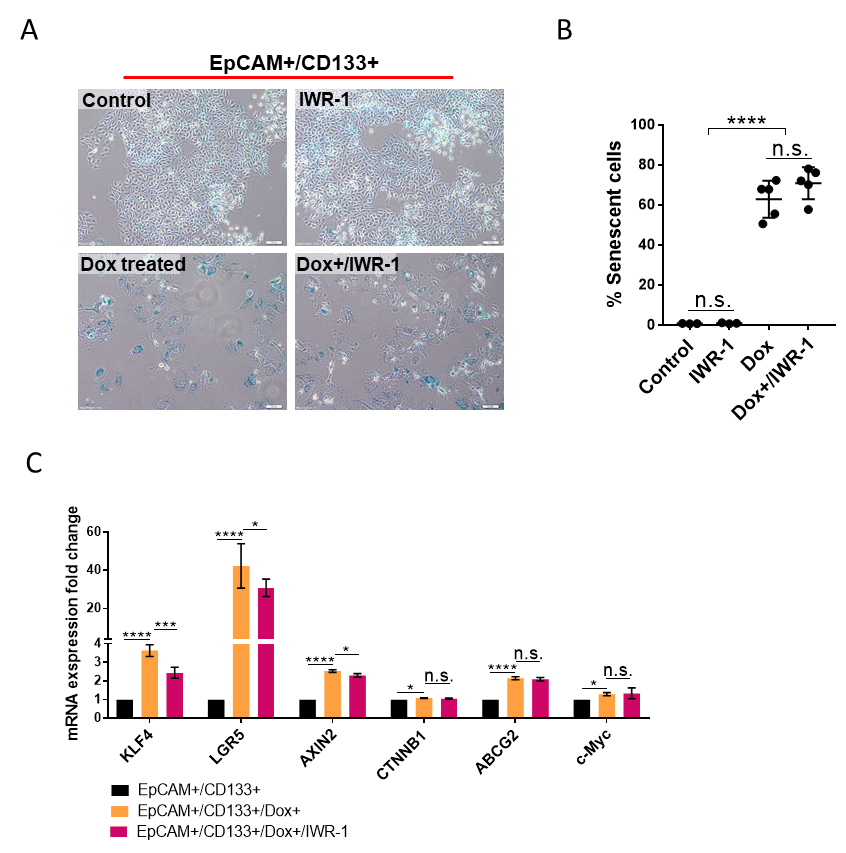


**Supplementary Fig. S1 The inhibition of canonical Wnt/β-catenin pathway reduces the expression of stemness- related genes in EpCAM+/CD133+ LCSCs.** (A) SA-β-gal staining of untreated and Dox treated EpCAM+/CD133+ LCSCs with and without IWR-1 treatment. (B) The number of senescent cells was graphed as percentage of the total cell number in all experimental groups. (C) The change in the expression of stemness related genes was analyzed by qPCR in all experimental groups. Data represent the average of at least three independent experiments. ">0.05"(n.s.), "≤0.05"(*), "≤0.01"(**), "≤0.001"(***)" ≤ 0.0001"(****)”. Error bars indicate standard deviation (SD).
